# Technique-Driven and Patient-Specific Variations in Complications of Mastectomy with Immediate Breast Reconstruction: An Evidence-Based Review

**DOI:** 10.3390/jcm15187334

**Published:** 2026-09-21

**Authors:** Lamorna Coyle, Gabrielle Odoom, You Jeong Park, Adam Siddiqui, Joseph A. Ricci, Neil Tanna

**Affiliations:** 1 Donald and Barbara Zucker School of Medicine at Hofstra/Northwell, Hempstead, NY 11042, USA; lcoyle1@northwell.edu (L.C.); jricci3@northwell.edu (J.A.R.); 2 Division of Plastic and Reconstructive Surgery, Northwell Health, Great Neck, NY 11021, USA

**Keywords:** mastectomy, immediate breast reconstruction, implant-based breast reconstruction, autologous breast reconstruction, hybrid breast reconstruction, acellular dermal matrix, DIEP flaps, patient selection

## Abstract

Mastectomy with immediate breast reconstruction (IBR) has become increasingly common, driven by advances in mastectomy techniques and the growing number of autologous, implant-based, and hybrid reconstructive options. The expansion of reconstructive strategies has introduced greater complexity in surgical planning and outcomes. Postoperative outcomes are influenced not only by mastectomy-related complications but also by complications specific to each reconstructive technique. Consequently, each combination of mastectomy and reconstructive modality carries unique benefits, limitations, and complication profiles that must be evaluated in the context of patient-specific factors, such as comorbidities, oncologic treatment, and reconstructive goals. This narrative review aims to characterize complication profiles associated with mastectomy and IBR to support improved perioperative management and individualized surgical care. To enhance clinical utility, this review includes representative clinical images that demonstrate variability in complication presentation across surgical approaches and patient contexts. This review presents a framework for understanding the factors that influence patient candidacy and outcomes in the setting of mastectomy with IBR, thereby supporting improved risk stratification and tailored treatment algorithms.

## 1. Introduction

Breast cancer is the most commonly diagnosed cancer worldwide and the leading malignancy affecting women [1,2]. In the United States alone, breast cancer accounts for approximately 32% of all new cancer diagnoses among women, and one in eight women (13%) will develop invasive breast cancer in their lifetime, with this number expected to climb to 38% by 2050 [3]. Given this existing disease burden and the anticipated rise in diagnoses, understanding the evolving landscape of surgical treatment for breast cancer is increasingly important.

Over the past two decades, rates of bilateral mastectomy have risen while rates of breast conservation surgery (BCS) have declined [4]. Several explanations have been proposed for this trend, including the expansion of genetic testing for hereditary breast cancer susceptibility. Women with risk factors such as BRCA1 or BRCA2 mutations or strong family history frequently elect to undergo prophylactic bilateral mastectomy, an option that reduces breast cancer incidence and mortality by more than 90% in high-risk patients [5,6]. The introduction of breast MRI, which can detect cancerous lesions that cannot be visualized on mammography, has also been implicated in this shift [4]. Bilateral mastectomy has gained popularity among those with unilateral breast cancer for both treatment and prevention even without genetic predisposition or oncologic risk factors [7]. As this surgical landscape evolves, a contemporary understanding of procedural risks and complications is necessary to guide patient counseling and surgical planning, especially considering the large role patient preference plays in surgical decision-making.

The decision to undergo mastectomy is closely linked to the option of reconstructive surgery. Up to 50% of women who undergo mastectomy also elect to undergo breast reconstruction [8,9,10,11]. Breast reconstruction rates have risen since the enactment of the Women’s Health and Cancer Rights Act (WHCRA) in 1998, which mandated insurance coverage for post-mastectomy reconstructive procedures in the United States [12]. After the enactment of the WHCRA, immediate breast reconstruction (IBR) rates nearly tripled to 40% from 1998 to 2011 [13,14,15,16]. In addition to the WHCRA, the expansion of nipple-sparing mastectomy techniques, coordinated interdisciplinary breast cancer care teams, and new autologous, hybrid, and implant-based reconstruction options have all contributed to the rise of IBR.

The psychosocial and quality of life benefits of IBR are well established in the literature. Compared to delayed breast reconstruction, IBR provides faster social reintegration and is associated with superior aesthetic outcomes, translating into improved patient self-esteem, body image, and sexuality [13]. However, the advantages of IBR must be weighed against the higher complication rate incurred when mastectomy and reconstruction are performed together, a risk that is only further compounded when there is an indication for postoperative adjuvant cancer therapy. Understanding this combined risk profile is highly important as mastectomy with IBR becomes more prevalent. Coordinated interdisciplinary care teams of medical and surgical oncologists, radiologists, and reconstructive plastic surgeons must therefore be aware of the array of complications associated with both mastectomy and different immediate reconstructive modalities. Such awareness is critical for patient-centered decision-making and perioperative management, as well as avoiding treatment delays.

This review synthesizes current evidence on complications associated with mastectomy and IBR, including those common to all IBR types and technique-specific issues for autologous, implant-based, and hybrid reconstruction techniques (Table 1).

The discussion raised by this review is particularly timely given the recent evolution of breast reconstruction practices. Advances such as perforator flap imaging and fascial-sparing donor-site harvests in autologous reconstruction, next-generation cohesive-gel implants and prepectoral placement trends in implant-based reconstruction, and the rise of hybrid approaches utilizing acellular dermal matrix (ADM) and other meshes have redefined reconstructive possibilities and, correspondingly, complication profiles. In the modern context of breast reconstruction, understanding the dynamic interplay between surgical technique and patient-specific factors is essential for developing a successful, individualized operative plan. This review aims to provide plastic surgeons and their multidisciplinary teams with a reliable framework for preventing, identifying, and managing postoperative complications associated with mastectomy and IBR, ultimately supporting the delivery of safer, tailored surgical care.

## 2. Overview of Immediate Breast Reconstruction Techniques

### 2.1. Autologous Breast Reconstruction

Autologous breast reconstruction techniques are popular options among women seeking a reliable, natural-appearing reconstructive outcome who are otherwise in good health. While health factors such as obesity or active smoking status are not considered absolute contraindications to autologous reconstruction, they remain important considerations when evaluating surgical candidacy [47,48,49]. The presence of adequate donor tissue volume relative to a patient’s native and desired breast size also dictates the feasibility of autologous reconstruction. The abdomen is often the first donor site assessed, as deep inferior epigastric perforator (DIEP) flap reconstruction is the leading option for autologous breast reconstruction due to superior tissue quality and reduced donor-site morbidity [49]. However, a paucity of abdominal tissue or a history of major abdominal surgery may preclude the use of DIEP flaps and necessitate reliance on alternative donor sites [50]. In these cases, options such as the profunda artery perforator (PAP) flap, transverse upper gracilis (TUG) flap, and lumbar artery perforator (LAP) flap may be considered. The absence of acceptable donor sites may rule out autologous reconstruction altogether. In addition to preoperative exam findings and patient preferences, donor site selection relies on preoperative imaging with magnetic resonance or computed tomography angiography to evaluate perforator size, location, and density.

### 2.2. Implant-Based Breast Reconstruction

Implant-based breast reconstruction remains the most common reconstructive modality, involving placement of either a silicone or saline implant in a single-stage or two-stage approach following tissue expansion [12]. It is often preferred because it is less invasive than autologous reconstruction, allowing for shorter operative times and quicker recovery periods. Implant-based reconstruction is also highly valuable for patients who do not meet candidacy criteria for autologous reconstruction [51]. Implant placement may occur in the subpectoral or prepectoral plane, with selection often guided by patient-specific factors and surgeon preference. Though the submuscular plane has historically been the most common choice for implant placement, prepectoral placement has risen in popularity in recent years [52]. With advancements in surgical techniques and implant technology, prepectoral implant reconstruction now achieves desirable aesthetic outcomes while avoiding the risk of animation deformity and morbidity associated with pectoralis muscle elevation [53]. Though some studies report that patients undergoing implant-based reconstructions have decreased long term satisfaction compared to those receiving autologous techniques, implant-based IBR continues to be the most popular reconstructive modality [54].

### 2.3. Hybrid Breast Reconstruction

Hybrid breast reconstruction techniques such as HyFIL^®^ (Hybrid Flap, Implant, Lipofilling) or HyPAD^®^ (Hybrid Flap, Prepectoral Acellular Dermal Matrix) combine autologous tissue transfer with implant-based methods, frequently incorporating exogenous materials such as small implants or ADMs to achieve greater volumetric enhancement and core projection than would be feasible with autologous tissue alone [55]. In a review by Heidemann et al. [56], the use of ADMs in immediate breast reconstruction was not associated with increased complication rates, suggesting that their integration may be safely employed within appropriately selected patient populations.

Selection of a reconstructive path is highly individualized and informed by each surgical patient’s own body habitus, comorbidities, oncologic factors, and aesthetic goals (Table 2).

While patient preference plays a central role in the process of shared decision-making, a patient’s ability to tolerate potential complications paired with the anticipated likelihood of such events influences the suitability of each reconstructive approach. An improved understanding of technique-dependent risks and the factors that modulate them is essential for personalizing operative planning and optimizing outcomes in reconstructive breast surgery.

## 3. Methods

A search of the PubMed/MEDLINE database from inception through July 2026 was performed to identify literature evaluating complications and outcomes of mastectomy with immediate breast reconstruction (IBR). The search combined controlled vocabulary (MeSH) and free-text terms across three concept blocks joined with the Boolean operator “AND,” while terms within each block were joined with “OR.”

(1) Block 1 (population/procedure): “mammaplasty”[MeSH] OR “breast reconstruction” OR “immediate breast reconstruction” OR “implant-based breast reconstruction” OR “autologous breast reconstruction” OR “DIEP flap” OR “TRAM flap” OR “PAP flap” OR “TUG flap” OR “LAP flap” OR “tissue expander” OR “acellular dermal matrix” OR “hybrid breast reconstruction.”
(2) Block 2 (mastectomy context): “mastectomy”[MeSH] OR “mastectomy” OR “nipple-sparing mastectomy” OR “skin-sparing mastectomy” OR “prophylactic mastectomy.”
(3) Block 3 (outcomes): “postoperative complications”[MeSH] OR “complication” OR “skin necrosis” OR “mastectomy flap necrosis” OR “nipple-areolar complex necrosis” OR “seroma” OR “hematoma” OR “surgical site infection” OR “wound dehiscence” OR “flap failure” OR “fat necrosis” OR “capsular contracture” OR “implant rupture” OR “implant extrusion” OR “implant malposition” OR “animation deformity” OR “red breast syndrome” OR “BIA-ALCL” OR “donor site morbidity” OR “abdominal bulge” OR “hernia.”

For each individual complication addressed in the review, a supplementary focused search was performed by pairing Block 1 and Block 2 terms with the specific complication term (e.g., “immediate breast reconstruction” AND “fat necrosis”; “implant-based breast reconstruction” AND “capsular contracture”) to ensure complication-specific literature was captured. Reference lists of included articles and relevant review articles were hand-searched to identify additional eligible studies.

Two reviewers (L.C. and G.O.) independently screened search results by title and abstract, followed by full-text review of potentially eligible studies. Disagreements were resolved through discussion and consensus. Eligible studies included those reporting relevant surgical complication or outcome data in adult women undergoing IBR following mastectomy. Non-English-language publications, case reports, editorials, and conference abstracts without an associated full-text article were excluded. Studies reporting exclusively on delayed breast reconstruction were not eligible. Emphasis was placed on studies published within the preceding five years, although older landmark studies were retained when they remained a primary source of evidence for a given topic.

Data were extracted by the same two reviewers, capturing study design, sample size, reconstructive modality, mastectomy technique, patient population, follow-up duration, complication definitions, and reported complication incidence. Because the included literature was methodologically heterogeneous, this work was conducted as a narrative rather than a systematic review, and no meta-analysis was performed. Accordingly, the incidence figures presented throughout this review represent the range of incidences reported across the included primary studies for a given complication and modality; they do not reflect formal statistical pooling, weighting, or calculation of summary effect estimates. Where a single representative or most frequently cited incidence is given, it is drawn directly from the source study and referenced as such.

No formal assessment of study quality or risk of bias (e.g., Newcastle-Ottawa Scale, Cochrane risk-of-bias tools) was performed, and no formal grading of the certainty of evidence (e.g., GRADE) was undertaken. Readers should therefore interpret the reported incidence ranges in the context of substantial heterogeneity among the included studies with respect to reconstructive technique, patient selection, follow-up duration, complication definitions, and treatment era. The principal conclusions of this review are predominantly supported by retrospective cohort studies, single-institution case series, and systematic reviews or meta-analyses of such studies, with a relative paucity of randomized controlled trials; this evidence hierarchy is acknowledged as a limitation and is discussed further in Section 8.

## 4. Complications of Mastectomy and Immediate Breast Reconstruction

### 4.1. Mastectomy Flap Ischemia and Skin Necrosis

The extensive dissection required in mastectomy often creates a predisposition towards skin flap ischemia, as injury to the breast vasculature during surgery disrupts blood flow to the remaining skin. The resulting pericapillary inflammation, along with increased tension and thinning, can threaten skin integrity and ultimately lead to necrosis. Ischemic changes are often observed in the lower pole of the reconstructed breast, as it experiences greater stretch and tension compared to the upper mastectomy skin flap [62]. The risk of skin or nipple areolar complex (NAC) necrosis is notably high in nipple-sparing mastectomy (NSM) due to the salvage of the entire native skin envelope, which creates increased tissue oxygen demand and may require longer periods of retraction in larger breasts [17,69]. The overall rates of mastectomy skin necrosis following NSM vary from 5 to 15%, while reported rates of nipple areolar complex (NAC) necrosis range from 2.1 to 7% [14,15,16,17,18,32,33].

**Figure 1:**
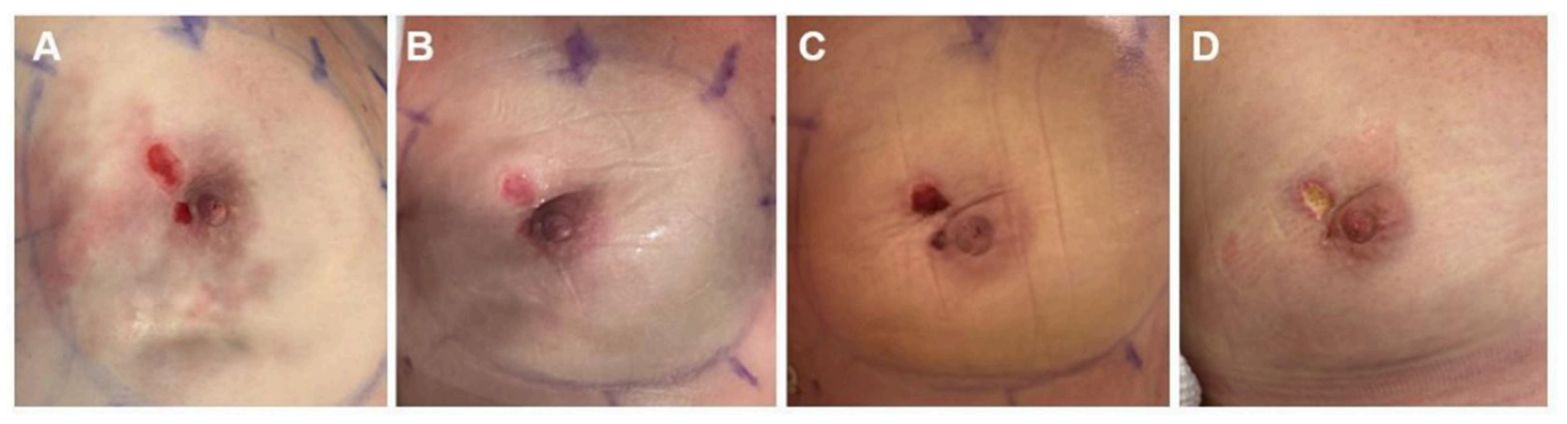
Progression of mastectomy skin ischemia after nipple-sparing mastectomy and IBR, from blister formation to development of an ischemic wound requiring in-office debridement. (**A**) Postoperative day (POD) 0; (**B**) POD 1; (**C**) POD 3; (**D**) POD 22.

**Figure 2:**
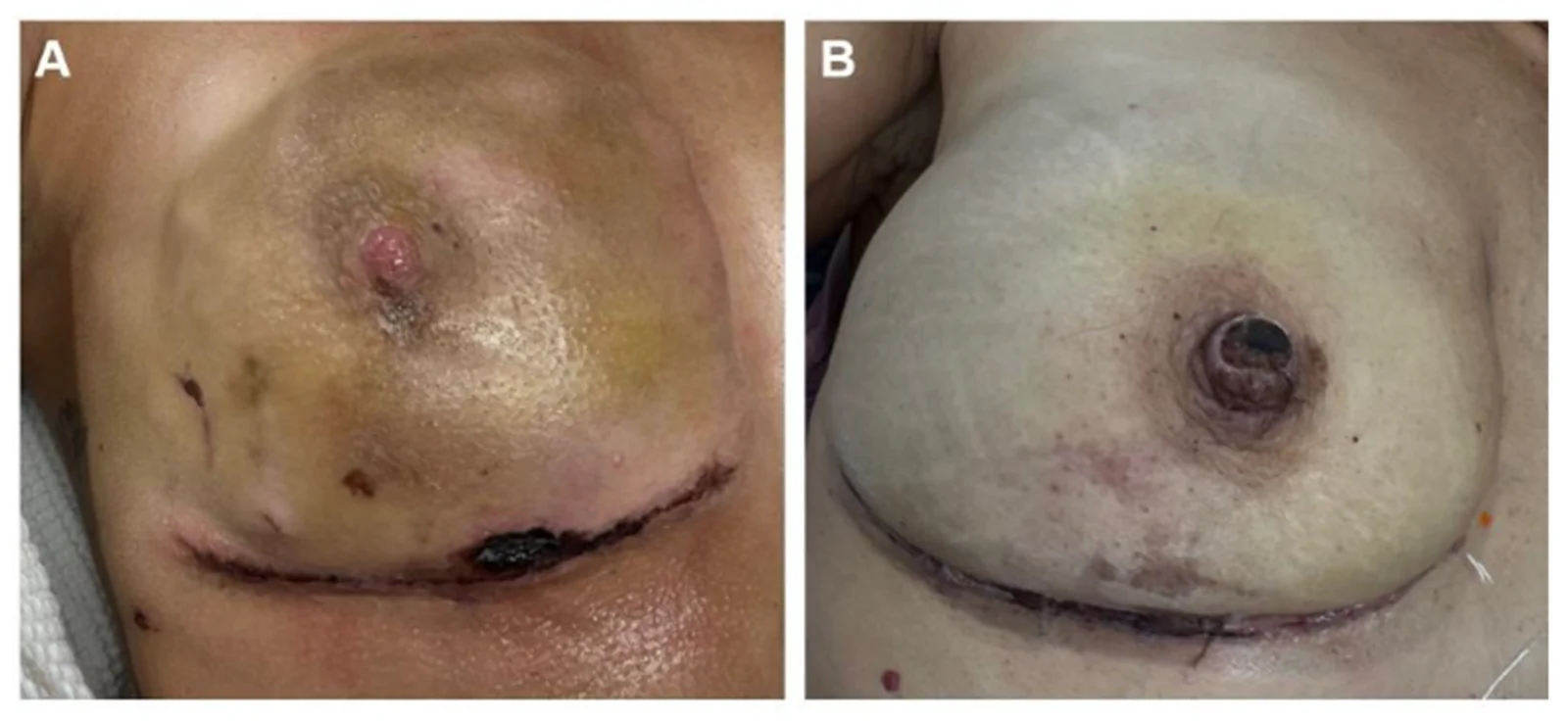
Examples of small areas of skin necrosis resulting from mastectomy-related ischemia requiring debridement. (**A**) Necrosis along inframammary fold incision following nipple-sparing mastectomy with immediate tissue expander reconstruction; (**B**) Necrosis present on inframammary fold incision and nipple-areolar complex after nipple-sparing mastectomy with immediate DIEP flap reconstruction.

**Figure 3:**
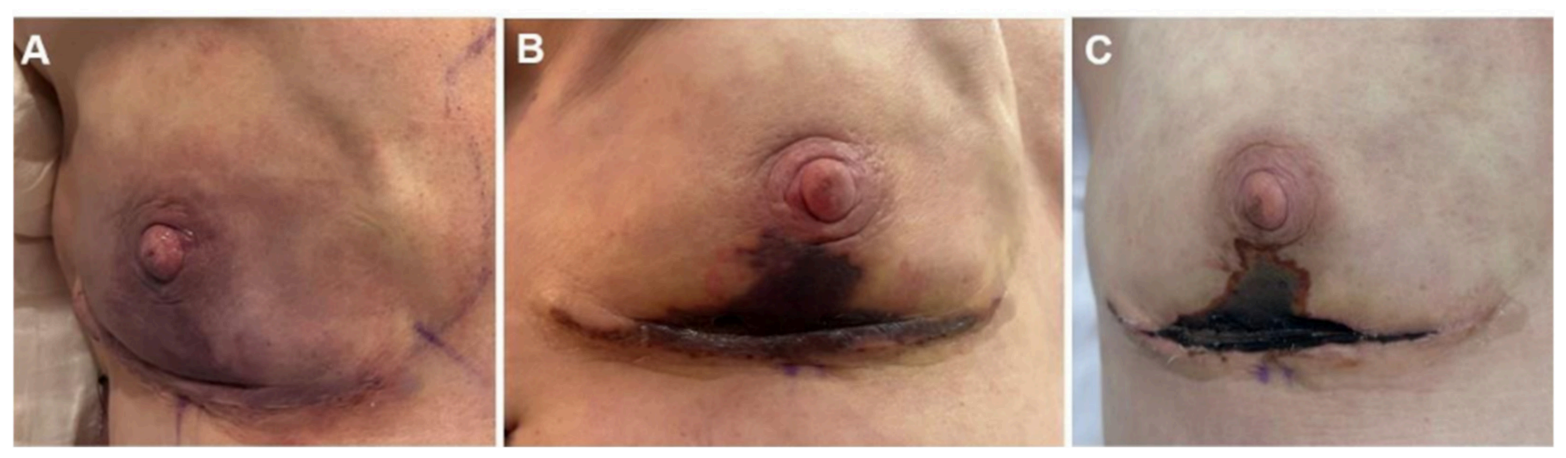
Progression of larger area of mastectomy skin necrosis following nipple-sparing mastectomy with immediate tissue expander reconstruction. (**A**) Early ischemia and venous congestion on POD 1; (**B**) Skin darkening and demarcation representing partial-thickness necrosis on POD 12; (**C**) Well-defined region of full-thickness mastectomy skin necrosis presents on POD 21, necessitating return to the operating room for excision and reclosure.

Increased risk of skin or NAC necrosis is associated with patient-specific factors such as smoking history, higher body mass index (BMI), history of radiation therapy, and more severe breast ptosis [16]. Surgical factors, including a greater volume of breast removed, periareolar incisions, and decreased mastectomy flap thickness may also augment the risk of NAC necrosis, though reconstructive modality also influences risk [70,71]. Conversely, lower mastectomy flap weights, inframammary incisions, and thicker mastectomy flaps may exert a protective effect [70,71]. Early ischemic changes may present as skin or NAC discoloration, delayed capillary refill, or blistering, which can progress to epidermolysis or full-thickness necrosis in severe cases (Figure 1). Mild, smaller ischemic areas <2 cm without device exposure may be treated conservatively with close monitoring and local wound care, while larger areas of full-thickness necrosis may require operative debridement to safely reestablish wound closure integrity (Figure 2 and Figure 3).

### 4.2. Skin Rash

Skin rash after mastectomy with IBR can arise from several different etiologies. Among these, contact dermatitis is the most frequent cause [62]. Contact dermatitis often manifests as localized pruritus and erythema with well-defined borders several days after exposure to a topical irritant, features that are often sufficient to establish a clinical diagnosis. Irritation primarily occurs in response to surgical dressings such as Tegaderm or Dermabond, or to sterile skin-prep products such as Betadine (Avrio Health L.P., Stamford, CT, USA) or ChloraPrep (Becton, Dickinson and Company (BD), El Paso, TX, USA) [22]. Risk factors for the development of skin rashes include advanced age, a personal or family history of atopy, history of radiation therapy, or prior reaction to topical agents [72]. Prevention begins with identifying any history of allergic sensitivities during preoperative evaluation. Inert topical agents like petroleum jelly may be superior to antibiotic ointments like Bacitracin or Neosporin, which may contribute to dermatitis [63]. Current recommendations may depend on the underlying cause but often consist of removing the offending agent or employing topical steroids, with most cases resolving within 2–4 weeks [73].

### 4.3. Infection

Infection can occur in all forms of mastectomy with IBR and carries significant consequences for postoperative management, aesthetic outcomes, and timely initiation of adjuvant oncologic treatment. Compared with mastectomy alone, mastectomy with IBR is associated with higher infection risk, with rates varying by reconstructive technique [74]. Reported infection rates for autologous breast reconstruction approximate 7–8%, with no significant differences observed across donor sites [19,20]. Infection rates are higher in hybrid reconstruction and highest in implant-only reconstruction, where reported incidence generally ranges from 5 to 14% [21,72,75]. Notably, infection rates in implant-based breast reconstruction exceed those observed in cosmetic breast augmentation, primarily due to the ischemic environment created by mastectomy and the larger surface area of exogenous material exposed to compromised tissue [76]. Gram-positive bacteria account for most implant-related infections, with the most common being *Staphylococcus aureus*, due to its high rate of cutaneous colonization, and *Staphylococcus epidermidis*, due to its strong affinity for foreign material and propensity for biofilm formation [77]. Non-tuberculous mycobacteria and *Cutibacterium acnes* represent atypical culprits in subacute or delayed breast pocket infections [78,79].

**Figure 4:**
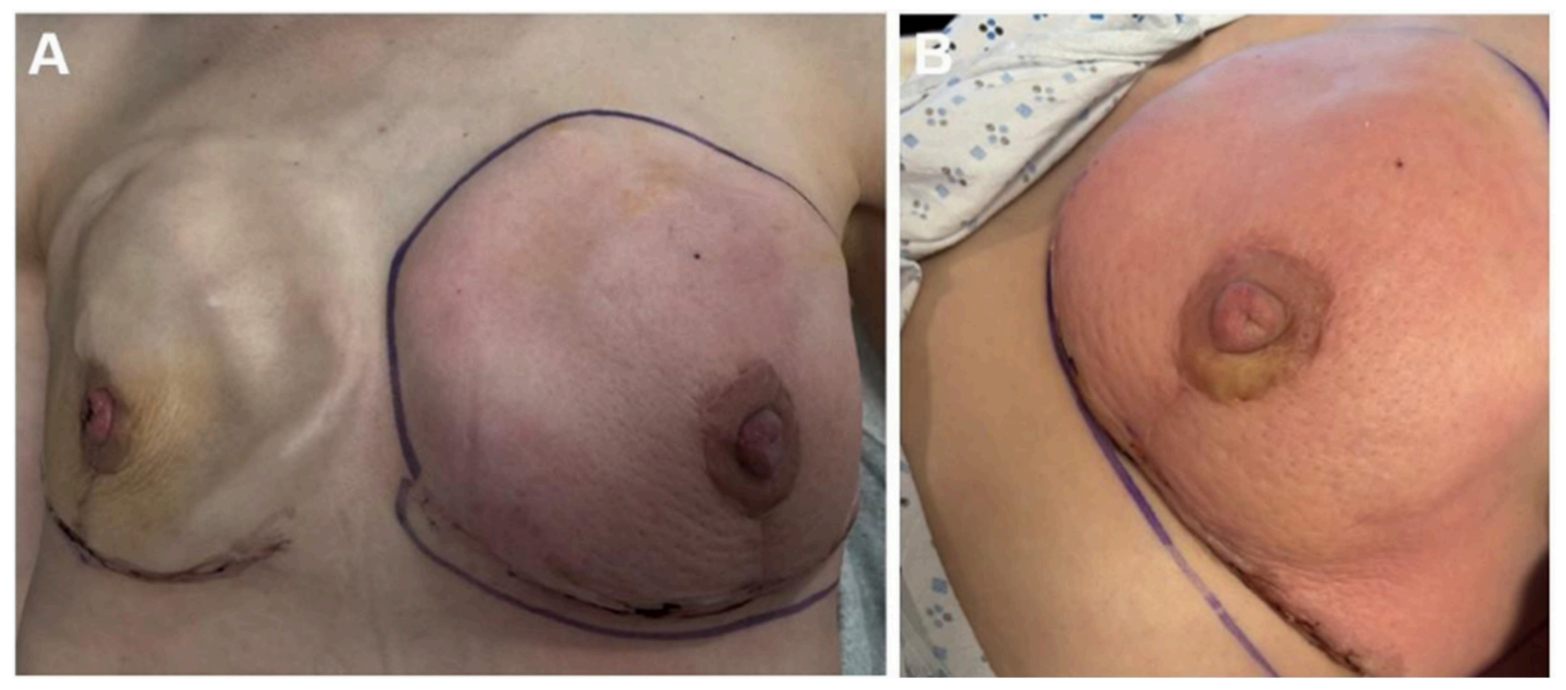
Left breast cellulitis after nipple sparing mastectomy with IBR using tissue expanders (**A**,**B**). Total breast erythema was accompanied by local pain and systemic symptoms. Patient required washout and removal of tissue expander. Fluid cultures grew *E. coli*.

**Figure 5:**
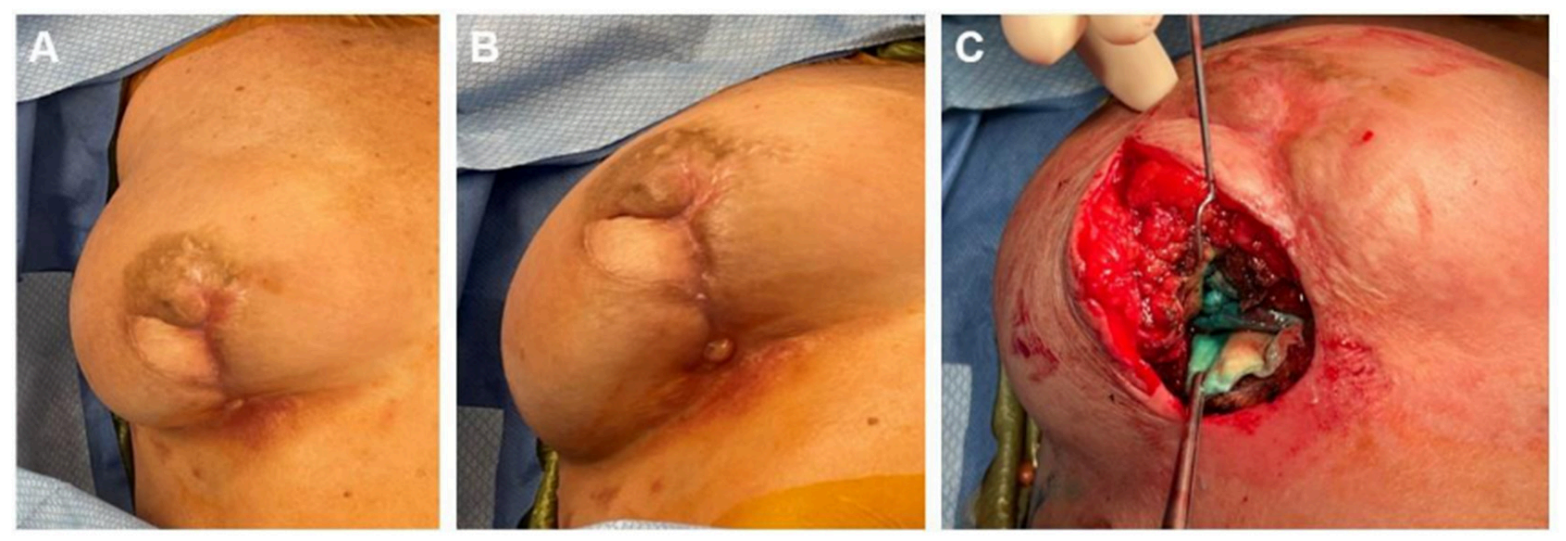
ADM-related complication following DIEP flap reconstruction with prepectoral ADM. (**A**,**B**) Erythema, rash, and drainage in inferior right breast area with exposure of the inferior margin of ADM; (**C**) Surgical exploration and removal of sinus tract was performed. Methylene blue dye injected into sinus tract diffusely stained the ADM, indicating an infected ADM surface. ADM removal and washout were performed.

Recognition of a surgical site infection requires careful clinical examination, as mild erythema due to infection may be difficult to distinguish from expected erythema in the early postoperative period. Common signs and symptoms of infection following mastectomy with IBR include breast redness, swelling, pain, purulent drainage from incision sites, and systemic symptoms such as fever (Figure 4). When infection is suspected, baseline laboratory tests, cultures of wound or drain fluid, and a breast ultrasound should be ordered to inform diagnosis and treatment [51]. Simultaneous initiation of oral or intravenous antibiotics (i.e., clindamycin, trimethoprim-sulfamethoxazole, ciprofloxacin, or vancomycin) with close monitoring is recommended [21]. Severe or persistent infections that occur in the presence of a foreign body (e.g., tissue expander, implant, or ADM) may indicate a mature biofilm matrix infection [74]. In such cases, explantation with washout is often necessary given the difficulty of eradicating infection without removing the device (Figure 5).

Across all forms of post-mastectomy breast reconstruction, key risk factors for infection include a history of preoperative radiation or chemotherapy, high BMI, advanced age, tobacco use, and the presence of other non-infectious complications such as hematoma or seroma [21,72]. The use of ADMs has also been variably associated with elevated infection risk. Standard prevention strategies include adherence to strict operative sterility and the use of perioperative prophylactic antibiotics [74]. In implant-based reconstruction, additional practices such as changing sterile gloves before implant placement, irrigating the surgical pocket with antibiotic solution, and using skin-contaminant mitigation tools such as the Keller funnel are widely used to reduce infection risk [51].

### 4.4. Wound Dehiscence

Wound dehiscence refers to a loss of closure integrity and is often a signal of poor wound healing. In some cases, it may signal more serious underlying complications, such as soft tissue infection [80]. Clinical presentation typically occurs 5–8 days postoperatively and may include bleeding or drainage at the surgical incision site, as well as separation of the skin edges.

The risk of wound dehiscence is influenced by factors related to both mastectomy and reconstruction, as well as patient characteristics. Reported rates of dehiscence are highest following mastectomy with immediate autologous reconstruction, followed by mastectomy with immediate implant-based reconstruction, and are lowest after mastectomy alone [32]. The incidence of wound dehiscence ranges from 3.5 to 14% in DIEP flap reconstruction and 1–5.1% for implant-based reconstruction [23,24,25,26]. Patient-specific factors, including older age, female gender, smoking, diabetes mellitus, chronic obstructive pulmonary disease, and obesity, have been associated with elevated wound dehiscence rates following breast reconstruction [23,80]. Operative characteristics such as incision type, wound closure technique, and prolonged operating times may also influence dehiscence risk [27].

Prevention of wound dehiscence focuses on minimizing tension on the suture line and optimizing closure integrity. Supportive garments, such as surgical bras and abdominal binders, may be used postoperatively to reduce tension on breast and donor site incisions. For high-risk patients, closed-incision negative pressure therapy (ciNPT) can be used prophylactically to support wound healing by promoting perfusion of local tissue and removing exudative fluid [27]. When wound dehiscence occurs, management must be guided by the severity of incisional separation. Mild cases of superficial dehiscence can be treated effectively with local wound care and regular dressing changes, while more significant incisional openings may benefit from ciNPT. However, separation of deeper fascial layers warrants immediate surgical intervention given the significant risk of evisceration. Evisceration should be managed with the application of sterile, saline-soaked dressings until primary closure can be achieved.

### 4.5. Fluid Collections

Mastectomy results in the formation of a cavity with impaired cutaneous blood flow and damaged lymphatics, creating ideal conditions for seroma or lymphocele development [68]. While a seroma is often defined as a buildup of exudative fluid attributable to an underlying inflammatory process, lymphocele represents an accumulation of lymph that may occur most often after injuring lymphatic vessels [81]. As such, the fluid contained in lymphoceles is frequently an opaquer tan-to-yellow color, which sometimes contains pathognomonic fat globules [81]. While seromas are contained by the borders of the surgical dead space, lymphoceles represent a form of pseudocyst that lacks a true epithelial lining [81]. Lymphoceles may persist longer than seromas, as injury to the lymphatic vessels may preclude symptom resolution for several months following surgery [81]. Most often these terms are used interchangeably in the literature or such that ‘seroma’ encompasses both types of fluid collections despite their distinct etiologies—a practice that will be upheld throughout this section.

**Figure 6:**
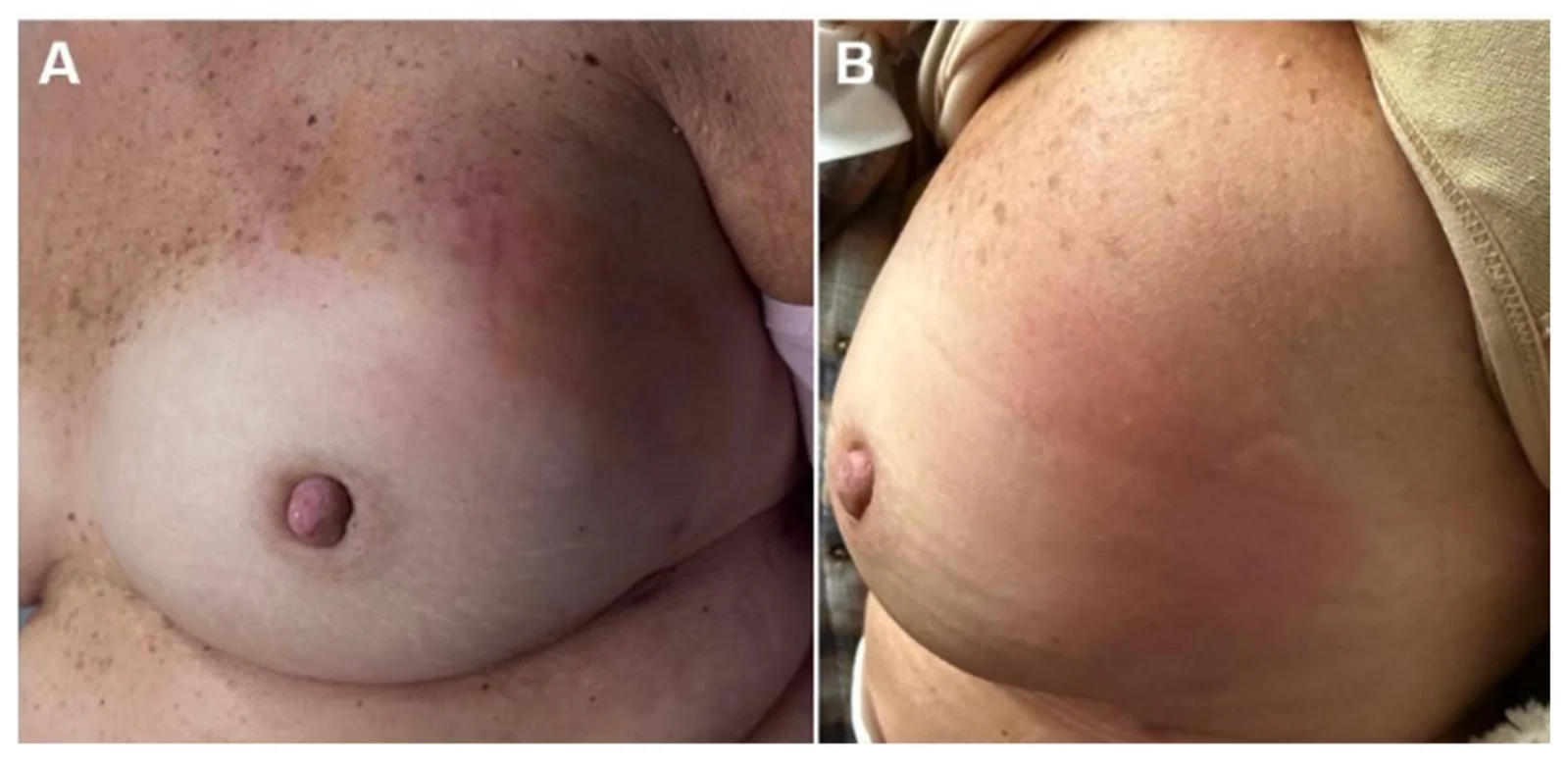
Left breast seroma after nipple mastectomy with immediate DIEP flap reconstruction, presenting with erythema, tenderness, and swelling of area with fluid collection (**A**,**B**).

Importantly, reported seroma rates are lower after mastectomy with IBR than after mastectomy alone, likely because reconstruction partially obliterates the dead space where fluid would otherwise collect [82]. Even so, seroma remains a relatively common complication of mastectomy with IBR, often presenting with breast swelling, erythema, and tenderness (Figure 6). The incidence of seroma ranges from 4.6 to 21% in autologous reconstruction and 2.5–8.4% in implant-based reconstruction [28,29,30,64,82,83]. Higher rates of seroma observed in autologous reconstruction are largely attributable to the added risk of donor-site fluid accumulation. In implant-based reconstruction, foreign materials such as implants or tissue expanders may promote fluid accumulation by triggering local inflammatory responses [83]. The use of ADMs may increase the risk of seroma formation, possibly due to delayed integration that leads to a cycle of exudative fluid buildup [30]. Across all reconstruction modalities, the extent of breast and axillary dissection, the number of lymph nodes removed, and the use of electrocautery are key factors that affect seroma and lymphocele risk [84]. Postoperative care and patient-specific factors, including drainage volume and timing, adherence to activity restrictions, and BMI, also contribute to the likelihood of fluid accumulation [67].

Prevention and early intervention are critical for seromas following IBR given the potential for downstream complications, including tissue ischemia from pressure-induced flap compromise and infection of the serous fluid collection leading to abscess. Standard preventative measures include the placement of closed suction drains to reduce dead space and monitor postoperative fluid quantity and quality, as well as activity recommendations during the early postoperative period. Recent evidence suggests a significantly lower rate of fluid collection in patients who perform range of motion (ROM) exercises 2 times daily compared to those who perform ROM exercises 0–1 times daily [85]. When seromas do occur, early percutaneous draining using a sterile technique is effective, and aspirated fluid should be sent for culture when infection is suspected.

### 4.6. Hematoma

**Figure 7:**
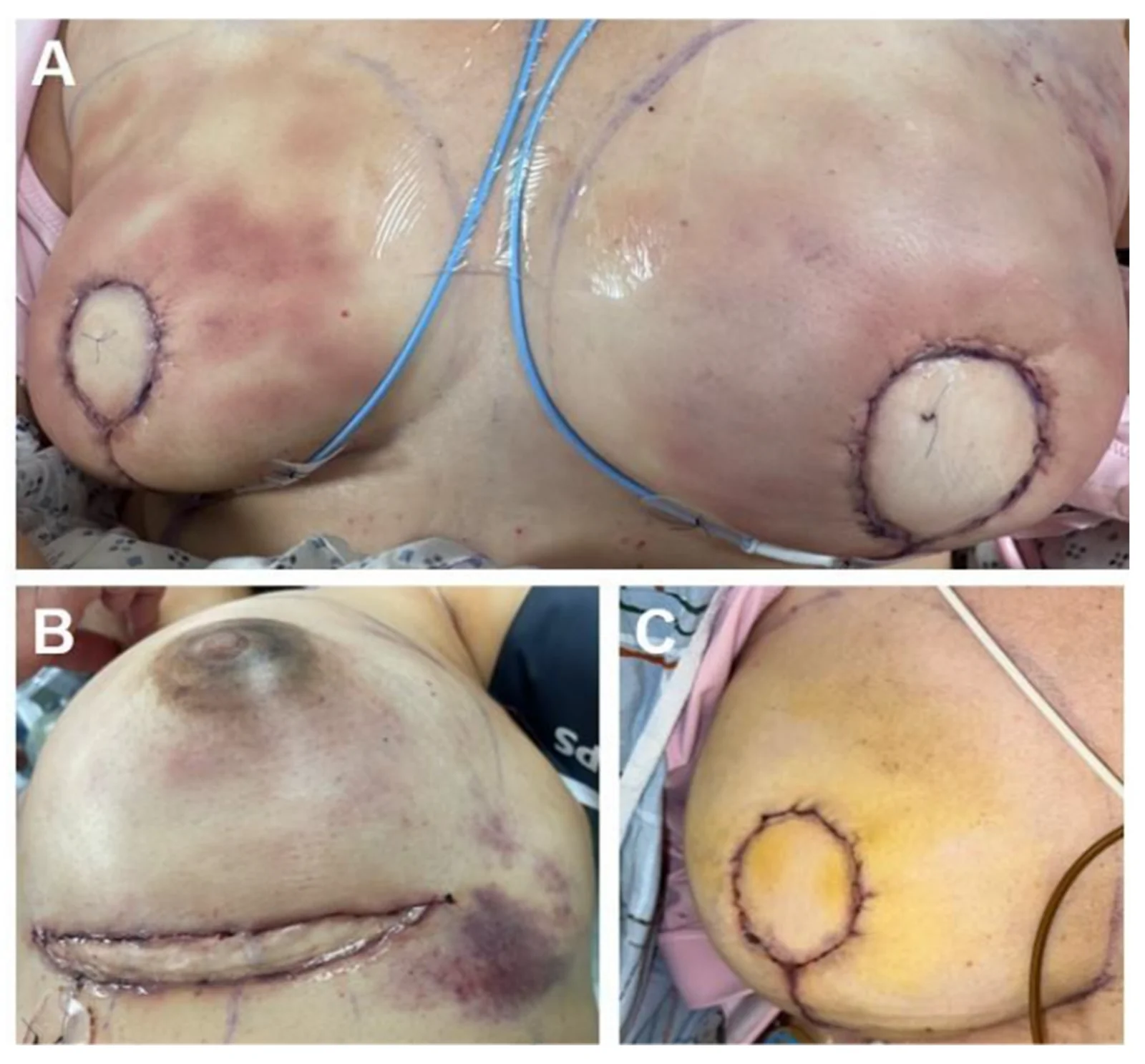
Cases of postoperative breast hematoma after nipple-sparing mastectomy with immediate DIEP flap reconstruction in three separate patients (**A**–**C**).

Hematoma formation following mastectomy and IBR typically occurs within the first 12–24 h after surgery and presents with breast enlargement, tenderness, swelling, and ecchymosis (Figure 7). While uncommon, late-occurring hematomas can result from trauma to the breast, coagulation disorders, early postoperative activity, and use of intraoperative corticosteroids [29,31].

Mastectomy with and without IBR appears to have similar rates of reoperation for hematomas based on a large-scale analysis of NSQIP data [86]. However, reported incidences of hematoma differ based on reconstructive modality, ranging from 1.8 to 9.4% for autologous reconstruction compared to 1–2.6% for implant-based reconstruction [31,32,33,34,35]. This discrepancy likely reflects the extensive vascular dissection at both the donor and recipient site during flap harvest and inset in autologous reconstruction, which requires extensive hemostasis to prevent postoperative bleeding. In implant-based reconstruction, it has also been hypothesized that larger implants may exert mechanical stress on blood vessels in the chest wall that contributes to hematoma formation [87].

Preoperatively, patients may be instructed to discontinue medications that raise bleeding tendencies, such as antiplatelet or anticoagulant agents, though any steps taken to mitigate hematoma risk must be carefully balanced against an indication for venous thromboembolism prophylaxis. Intraoperatively, extensive hemostasis is essential for hematoma prevention, and the use of tranexamic acid (TXA) has been shown to significantly reduce hematoma rates when administered intravenously or topically [21]. While closed suction drains do not appear to lower hematoma risk, they facilitate early recognition of a developing hematoma by allowing outflow of continued bleeding and reduce the likelihood of blood clot formation in the breast pocket. Management of hematomas typically requires return to the operating room for breast pocket exploration, hematoma evacuation, and hemostasis [21,31]. Prevention and early treatment are critical, as hematoma development is associated with an increased rate of capsular contracture in implant-based reconstructions and, like seroma, can lead to pressure necrosis of the surrounding tissue and concomitant infection of the blood pocket.

## 5. Complications Specific to Autologous Breast Reconstruction

### 5.1. Flap Failure

Flap failure is one of the most feared complications in microsurgical breast reconstruction and typically results from venous or arterial compromise following free tissue transfer [36,88,89]. Flap salvage is generally less successful in cases of arterial compromise, as the resulting hypoxia and accumulation of toxic reactive oxygen species can induce irreversible microvascular damage. Flaps affected by venous compromise may be more amenable to salvage; however, tissue damage and collapse of the microvasculature can result from impaired venous outflow. Risk factors for flap failure include hereditary disorders of collagen formation, coagulopathies, small vessel caliber, obesity, smoking history, and prolonged operating time [61,88,89]. Overall flap failure rates have been variably reported as 0.5–5%, depending on flap type and degree of necrosis [29,30,36].

Ischemic risk is the greatest at the distal edges of flaps, typically furthest from chosen perforators. Preoperative MR or CT angiography can aid in ideal perforator selection by mapping vessel size and location. Intraoperative technologies such as indocyanine green fluorescence angiography and Doppler ultrasound can also assist with perforator selection, assessment of tissue perfusion during flap harvest, and detection of early venous congestion or ischemia after anastomosis [65,90,91]. The use of 2–3 perforators based off either the medial or lateral perforators has been suggested as the optimal number for sufficient perfusion. Supercharging, a microvascular technique to augment venous outflow via connection of an additional vein, has also demonstrated reduced rates of operative take-back [65].

**Figure 8:**
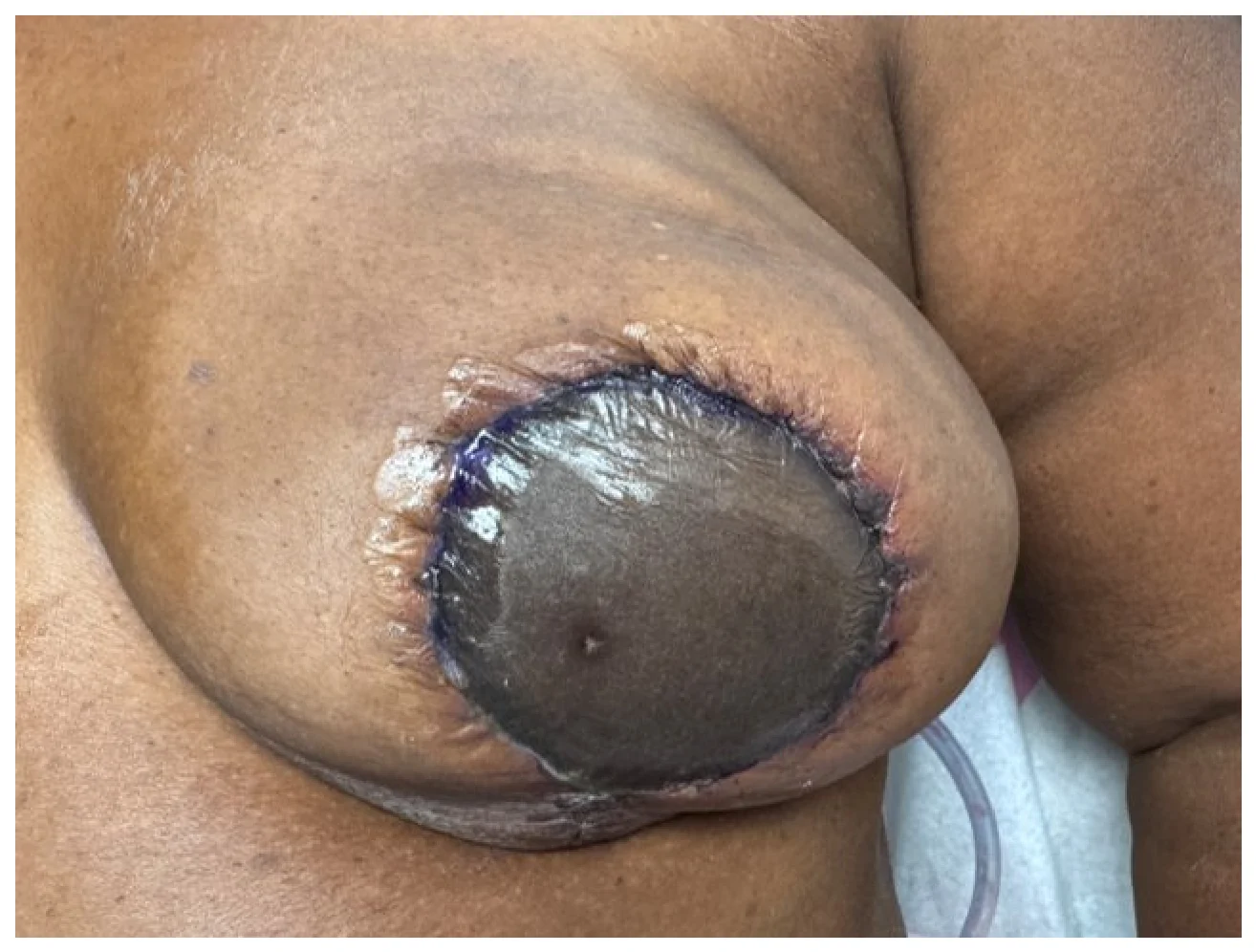
Total flap loss following skin-sparing mastectomy with immediate DIEP flap reconstruction. Flap nonviability was indicated by discoloration and lack of perfusion of abdominal skin paddle, as well as coldness and firmness of flap, necessitating return to the operating room for flap removal. If thrombosis is discovered upon exploration, intra-arterial tissue plasminogen activator may be used for direct fibrinolysis, or systemic heparinization may be performed for 4–7 days post-takeback. Though there is currently no high-level evidence that establishes an optimal systemic anticoagulation regimen in this setting, the 4–7 duration of heparinization is firmly anchored to the six-day period required for neoendothelialization of the fresh anastomosis [37].

Early recognition of perfusion problems is essential, as timely intervention within the ‘golden window’ of 6–12 h can prevent irreversible tissue loss. Clinical indicators of flap necrosis include progressive discoloration, coolness, increasing pain, and the development of nonviable or open wounds within the flap skin paddle. Total flap loss necessitates return to the operating room for removal of the nonviable flap, followed by several months of recovery prior to additional breast reconstruction surgery (Figure 8). Partial flap loss, on the other hand, may be amenable to salvage through hyperbaric oxygen or conservative wound care [29].

### 5.2. Fat Necrosis

Fat necrosis is a common complication following autologous breast reconstruction, with a reported incidence ranging from 3.0 to 37.9% according to a systematic review of 70 articles covering abdominal flap-based breast reconstruction techniques [92]. Following autologous transfer, fatty tissue integrates into recipient tissue in a process analogous to skin graft revascularization: imbibition, inosculation, and neovascularization. Complications with this process can lead to inadequate perfusion of transferred fatty tissue, triggering a sterile inflammatory reaction to necrotic adipocytes that results in fat necrosis [93]. On ultrasonography, the progression of fat necrosis can be visualized in three distinct stages: the hyperacute phase (hyperechogenic), the inflammatory phase (partially formed oil cyst with fibrotic remnants), and the fibrotic phase (nearly complete oil cyst with minimal fibrotic tissue) [93,94,95].

While its clinical presentation can vary, fat necrosis most often appears as a palpable, firm mass that can be painful and aesthetically undesirable [92,96]. Fat necrosis typically resembles a calcified oil cyst on MR imaging but can present as a complex solid mass on ultrasound, necessitating a multimodal process for diagnosis [95]. Moreover, it frequently mimics breast cancer on physical examination and radiologic imaging, which can complicate postoperative surveillance. On post-treatment MRI, dynamic contrast enhancement or washout raises concern for recurrence, while a non-enhancing central fat signal typically raises the likelihood of benign fat necrosis [94]. Pathologic confirmation is warranted whenever irregular contour or spiculated calcifications are present and may be necessary.

Risk factors for fat necrosis are similar to those of flap failure, as both are rooted in inadequate perfusion. Contributing factors include pre- and postoperative radiation therapy, greater flap weight, and previous abdominal scarring [96,97]. Sufficient perforator selection and intraoperative supercharging are potential strategies for mitigating fat necrosis risk [37,65]. Conservative treatment with NSAID use and local massages is often effective for resolving mild cases. However, larger or more symptomatic areas of fat necrosis may necessitate surgical removal [96].

### 5.3. Donor Site Complications

**Figure 9:**
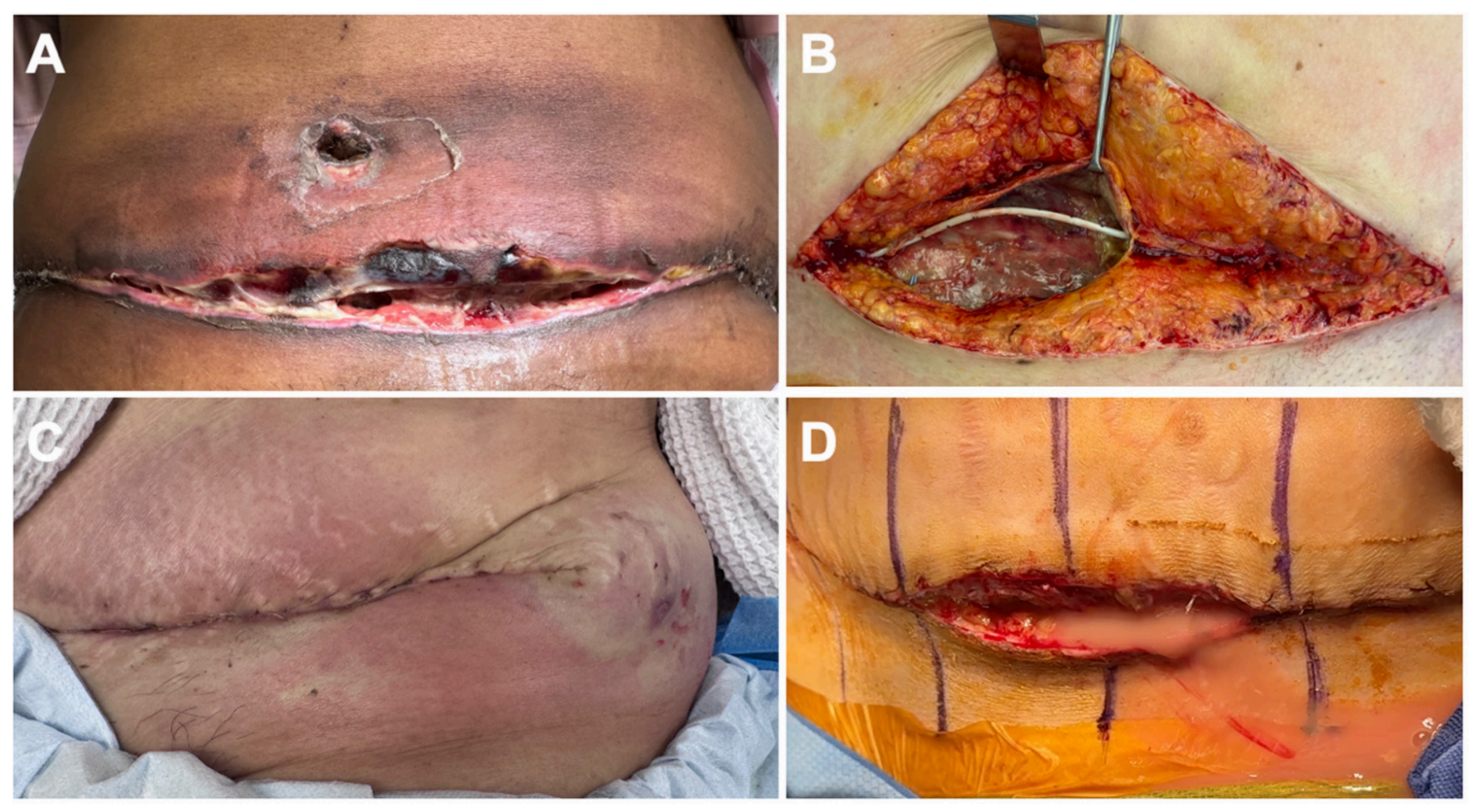
Examples of donor site complications. (**A**) Abdominal donor site wound dehiscence following DIEP flap breast reconstruction; (**B**) Abdominal donor site seroma following DIEP flap breast reconstruction; (**C**) Abdominal donor site infection following DIEP flap breast reconstruction; (**D**) Abdominal donor site infection with abscess following DIEP flap breast reconstruction.

Complications arising from autologous tissue transfer are unique in that there are two surgical site locations (the donor and recipient site) that may experience obstacles to proper wound healing. Both donor and recipient sites share many of the same susceptibilities, such as wound dehiscence, infection, seroma, hematoma, and skin necrosis (Figure 9).

Each donor site also remains vulnerable to site-specific morbidity, with abdominal hernia being a notable donor site challenge in DIEP flap reconstruction. Though the DIEP technique largely spares the abdominal musculature, variable degrees of muscle dissection may be required depending on perforator branching patterns. Such dissection can weaken the integrity of the abdominal wall and predispose the patient to hernia. Reported incidences of abdominal hernia following DIEP reconstruction are variable but generally range from 0.7 to 4.9% [26,30,38]. Additional factors can exacerbate the risk of abdominal hernia or bulge formation following DIEP flap reconstruction, including patient factors such as obesity and excessive coughing [25]. Harvesting two or more perforators and perforator laterality are also risk factors for abdominal hernia after DIEP reconstruction [98].

Several techniques for prevention and management of abdominal hernia or bulge have been described for use in DIEP reconstruction, including the use of reinforcing meshes for fascial closure [38,99]. DellaCroce et al. [100] describe a novel approach for optimizing the balance between robust perforator selection and reduced abdominal site morbidity with the abdominal perforator exchange (APEX) flap. APEX spares the intervening rectus fibers between perforators by adding an additional anastomosis to reconnect the medial and lateral divisions of a split perforator, while maintaining a flap failure rate of 0.27% [100]. Additionally, APEX and other fascial-sparing techniques may mitigate neuropathic pain symptoms often experienced by patients who undergo abdominally based free flap reconstruction [100]. In terms of management, Haddock et al. [38] devised a treatment algorithm that classifies postoperative hernias into three distinct types: (1) abdominal weakness, (2) smaller unilateral abdominal bulge, or (3) true hernia or bilateral abdominal bulge. Depending on severity, treatment recommendations may include physical therapy or surgical repair with mesh.

## 6. Complications Specific to Implant-Based Breast Reconstruction

### 6.1. Implant Extrusion

Implant extrusion is a relatively uncommon complication that occurs when breakdown of the overlying tissue results in exposure of the implant, often triggered by infection or wound healing issues. Prepectoral implant placement has historically been associated with higher rates of skin necrosis and implant extrusion due to the absence of extra padding and support provided by the pectoralis major in the submuscular approach [101]. However, advances in implant-based IBR, such as ADM use and polyurethane-coated silicone implants, have helped mitigate these concerns and support the growing popularity of prepectoral implants in reconstruction. Lipofilling has also been used successfully to enhance cushioning over the implant and decrease extrusion risk [102,103,104]. Clinical adjunctive tests such as the pinch-test threshold have been used with success to determine adequate soft-tissue thickness. The rate of implant exposure in implant-based reconstruction techniques is low, variably reported between 0.25 and 8% [42]. Risk factors for implant extrusion include older age due to skin and tissue thinning and subsequent breakdown, as well as post-mastectomy radiation. Treatment for implant extrusion includes complete implant removal followed by delayed implant replacement or autologous reconstruction if patients prefer further reconstruction [42,105,106,107].

### 6.2. Red Breast Syndrome (RBS)

RBS is a rare inflammatory condition that occurs in the setting of ADM-based breast reconstruction. RBS is characterized by localized erythema corresponding to the borders of the underlying ADM and can be difficult to distinguish from cellulitis [108]. However, classic signs of infection, such as warmth, tenderness, and swelling, are typically absent in RBS [108]. While the exact pathophysiology of RBS is incompletely understood, several potential mechanisms have been described. One theory is that RBS represents a type IV hypersensitivity reaction triggered by DNA remnants within ADM [109,110]. Others speculate that impaired lymphatic drainage caused by mastectomy contributes to RBS, and that the use of ADMs with rapid recellularization and revascularization may promote prompt symptom resolution [110]. Radiation therapy has also been reported as a risk factor for RBS, though evidence for this association remains limited [111]. In addition to uncertainty surrounding the etiology of RBS, there is no consensus on optimal prevention and treatment methods. One prevention method reported by Nahabedian et al. [111] involves washing ADM with sterile saline and then an antibiotic solution prior to implantation. Monitoring inflammatory markers such as C-reactive protein or procalcitonin, as well as radiographic imaging with ultrasound, may be helpful in ruling out periprosthetic fluid collection that may masquerade as an autoimmune phenomenon. A range of treatment strategies have been described, including management of RBS with corticosteroids or antibiotics [111]. However, in refractory cases, surgical intervention with ADM washout or removal may be required to achieve complete symptom resolution [111].

### 6.3. Implant Malposition

Implant malposition is a common occurrence that can compromise aesthetic outcomes in implant-based IBR, affecting an estimated 9.8% of breasts after implant-based reconstruction [46]. The degree and direction of implant displacement are often influenced by surgical plane dissection, resulting implant pocket size, implant type, presence of capsular contracture, and patient body habitus. Textured prostheses were initially developed to combat implant malposition; however, they have since fallen out of favor due to their association with BIA-ALCL. Pacifico et al. [112] developed an implant malposition classification system that uses the natural breast footprint and ideal aesthetic lines to determine the degree and direction of displacement. Correction of implant misalignment is often tailored to the defect and may involve placement of internal or external sutures, relocation of the implant to a new surgical plane (e.g., dual-plane placement), or providing extra support using capsular flaps or ADMs. Capsulorrhaphy or capsulectomy may also be warranted to tighten or adjust the capsule surrounding the implant.

### 6.4. Animation Deformity

Animation deformity refers to the contraction of the overlying pectoralis major muscle against the breast implant, resulting in unnatural breast movement or displacement during muscle engagement. When implants are placed in a submuscular pocket, muscle contracture during chest wall movement results in superolateral implant displacement and extreme compromise of aesthetic outcomes in 75–100% of cases [44,113]. Prepectoral placement generally avoids this risk, though the extra tissue thickness and vascular protection afforded by the pectoralis major muscle is lost. The implementation of fat grafting and ADMs has improved some of the cushioning deficits encountered in prepectoral implant placement while also minimizing rippling and implant palpability [45].

### 6.5. Implant Rupture

Rupture occurs in approximately 10–15% of implants within 10–15 years following placement, with clinical presentation varying by implant type [41]. Saline implant rupture results in an abrupt breast volume change that is apparent on physical examination alone, as saline leaks rapidly and is quickly absorbed by surrounding tissue. By contrast, silicone implant rupture can cause subtle or progressive changes in volume that may not be identifiable at all until routine mammography is performed [21]. Silicone implant leakage is typically intracapsular, though extracapsular extravasation can also occur and lead to a more pronounced clinical presentation. For this reason, ultrasound or MRI is recommended beginning 5–6 years after initial placement of silicone implants and every 2–3 years thereafter for asymptomatic patients [114]. Higher rates of implant rupture are associated with older implant age, capsular contracture, and subglandular implant placement. Double-lumen and polyurethane-coated implants, as well as implants manufactured by McGhan, have demonstrated lower rates of rupture compared to smooth gel implants and Surgitek [114]. Management of implant rupture depends on implant type. In the case of saline implant rupture, early implant replacement is recommended to preserve implant pocket size [21]. When silicone implant rupture is detected, implant removal or replacement with capsulectomy is often preferred to prevent any local or systemic inflammatory reactions.

### 6.6. Capsular Contracture

Capsular contracture is the most common complication following implant-based reconstructive and aesthetic breast surgery and is a frequent reason for reoperation [66]. Estimated incidence of capsular contracture following implant-based reconstruction generally ranges from 10 to 25% [39,40]. Though the underlying mechanisms remain unclear, capsular contracture is believed to be a result of an exaggerated immune response to the presence of a foreign body in the breast pocket. Prolonged inflammatory reactions to silicone may play a role in contracture etiology, as light microscopy evaluation of capsules from severe cases has revealed significant silicone dispersion that is absent or minimal in capsules from milder cases [66,115]. Clinical presentation of capsular contracture tends to include breast implant hardening, distortion, and pain. Severity is traditionally classified with the Spear-Baker grading scale, though in recent years several other methods have risen in popularity [115,116].

In a 2023 retrospective chart review of 340 direct-to-implant reconstructions, higher rates of capsular contracture were associated with older age, increased BMI, smaller implant size, and adjuvant radiotherapy [39]. In the same cohort, skin-sparing mastectomy was associated with the lowest rate of capsular contracture, followed by nipple-sparing mastectomy and total mastectomy. Evidence comparing the contracture risks of saline versus silicone implants is unclear; however, studies have indicated that smooth round implants may be more protective against capsular contracture compared to textured implants. Several operative strategies have been described for reducing capsular contracture risk, including pocket irrigation with triple-antibiotic solution, subpectoral implant placement, and wrapping implants with ADM or other meshes [39,66,99].

The severity of capsular contracture dictates appropriate management. Surgical options include capsulotomy, partial capsulectomy, and total capsulectomy. While capsulotomy and partial capsulectomy are quicker and less invasive operations, leaving behind capsule remnants may predispose patients to recurrence [114]. Total capsulectomy with implant exchange is considered the gold standard for severe or recurrent capsular contracture, and incorporating ADM during revision procedures may further mitigate recurrence.

### 6.7. Breast Implant-Associated Anaplastic Large Cell Lymphoma (BIA-ALCL)

The first reports of BIA-ALCL date to 1999, when Keech et al. published the discovery of a novel T-cell lymphoma associated with breast implant capsules [117,118]. BIA-ALCL was later inaugurated by the WHO in 2016 as a new type of lymphoma, with the only known risk factor for development being macrotextured breast implants, though cases have also been described with smooth implants [119]. Estimated incidences of BIA-ALCL vary, ranging from 0.0025% to 0.18% among patients with textured breast implants [43]. The average time to presentation after implant placement is 7–9 years, with a delayed seroma (>1 year postoperatively) as the most common presenting symptom [43]. Cases of BIA-ALCL have been reported in both cosmetic and reconstructive breast implant capsules, and most hypotheses suggest that the underlying pathophysiology is a chronic inflammatory reaction to foreign material [120]. Current treatment standards include implant removal with en bloc capsulectomy, coupled with chemotherapy, immunotherapy, or radiation in advanced cases involving lymph node infiltration or distant metastasis. Mortality rates are approximately 2.8% in the first year after diagnosis [121].

Instances of breast implant-associated squamous cell carcinoma (BIA-SCC) have also been reported in the literature, though cases have not been linked to specific implant characteristics. In contrast with BIA-ALCL, BIA-SCC is very rare, with only 19 documented cases globally, and presents at an average of 22 years after initial implantation. This extremely low incidence has impeded the development of standardized diagnostic and therapeutic guidelines for the disease, which carries an exceedingly high mortality rate of 48% at six months [117].

## 7. A Guide to Individualized Planning for Breast Reconstruction

**Figure 10:**
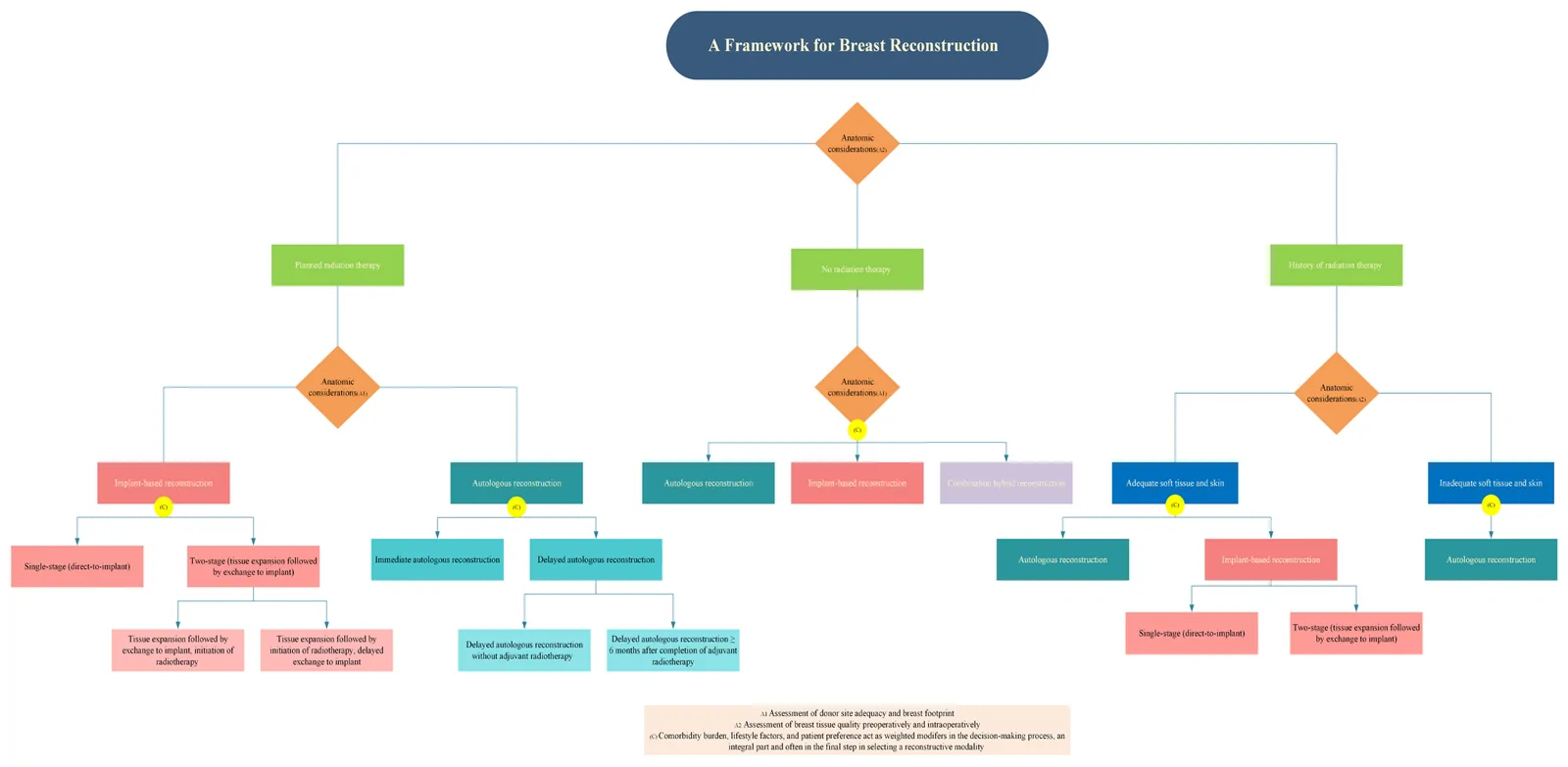
Flowchart for individualized planning in breast reconstruction in the setting of previous or planned post-mastectomy radiation therapy [123].

Given the diversity in breast reconstructive techniques and the associated differences in complication profiles, individualized surgical planning requires a structured algorithm to produce an effective blueprint for future operative intervention. Considering the undue influence of adjuvant radiotherapy on reconstructive outcomes, establishing a history of PMRT or a plan for future irradiation should be the first step in formulating an appropriate reconstructive framework, followed by anatomical considerations. Guidelines from the National Comprehensive Cancer Network provide explicit timeline and staging recommendations for both implant-based and autologous techniques (Figure 10) [122].

As radiotherapy significantly increases the baseline risk of capsular contracture, malposition, implant exposure, infection, and reconstructive failure, autologous approaches are often preferred when technically and anatomically feasible. The options for direct-to-implant approaches and immediate autologous reconstruction remain for patients with a need for PMRT, though the balance is shifted towards delayed and staged approaches. Direct-to-implant candidacy is often evaluated through preoperative breast size, desired postoperative size, tissue quality, and the presence of additional risk factors. Regarding complication rates, recent evidence suggests favorable outcomes in direct-to-implant approaches that mirror or exceed those seen in staged tissue expander reconstruction [122,123]. Since radiation increases the risk of fat necrosis and poor cosmesis, immediate autologous approaches are generally avoided when PMRT is planned, with most practitioners favoring a delayed intervention at least 6 months after completion of PMRT [122]. Still, immediate reconstruction remains a viable option, with several studies in similar contention over whether the timeline of autologous reconstruction significantly impacts complication rates [122,123,124,125,126].

After eliciting oncologic history and determining the precise role of radiation therapy, the choice between staged implant-based or autologous approaches is frequently governed by the body habitus and prior surgical history. Sufficient abdominal tissue often makes autologous reconstruction using DIEP flaps the preferred approach, though secondary sites make TUG, PAP, and LAP flaps acceptable secondary choices. In the absence of sufficient tissue at any of these donor sites, implant-based reconstruction becomes the default option [122]. Other characteristics such as breast ptosis and macromastia may require a reduction-pattern approach with subsequent implant placement or flap-based reconstruction [122].

A thorough history should identify comorbidities, demographic variables, and lifestyle factors that may elevate the risk of certain complications (Table 2). After elucidating which patient-specific risk factors may influence postoperative outcomes, the salience of each comorbidity should be weighed against the inherent complication burden of each reconstructive approach (Table 1). A machine learning model built to predict both implant-based and autologous breast reconstruction complications identified smoking, adjuvant radiotherapy, BMI, age, and diabetes as the top predictors of 1-year complication rate (XGBoost AUROC 0.83) [123]. While comorbidities are rarely an absolute contraindication to reconstructive approaches, they do act as an important weighted modifier in the decision-making process.

Beyond relative risk-benefit discussions that account for complication predispositions, individualized wound-complication risk estimates may also be performed to provide a quantifiable patient-specific risk assessment. In a separate risk prediction model for wound complications (optimism-corrected C statistic 0.735), immediate reconstruction, bilateral mastectomy, BMI, depression, and active smoking status were all correlated with severe wound complications, though autologous flaps were less represented in this study [32]. While some patient-specific variables may prove alterable, diligent preoperative counseling is an essential component of surgical preparation and patient compliance with preoperative recommendations carries the potential to shift surgical candidacy. For example, in a patient with a history of adjuvant radiotherapy and diabetes, the risks of delayed wound healing, infection, and anastomotic failure are elevated in an autologous approach, though this technique may also provide great cosmetic and functional benefit by recruiting healthy bulk to provide coverage in a previously irradiated region. Special care should be taken to maximize glycemic control prior to any intervention (ideally <200 mg/dL with an absence of urinary glucose), while weight optimization may also be necessary to ensure safety and reduce surgical risk [57]. Through careful monitoring and multidisciplinary care, this patient may modify their surgical eligibility, becoming more apt to a flap-based reconstructive option. Similarly, though active smoking status often disqualifies patients from reconstruction, patients who are amenable to smoking cessation at least 4 weeks prior to surgery and 2 weeks postoperatively reduce their odds of complication and may alter their qualification. Finally, an assessment of a personal or family history of thrombophilia is an imperative step in preoperative planning, as this may significantly limit options for autologous reconstruction [127].

Lastly, the patient’s own reconstructive goals should guide technique selection. An open, structured discussion, ideally incorporating a validated assessment tool such as the BREAST-Q patient-reported outcome framework, should take place to establish expectations regarding procedure length, recovery time, aesthetic results, and the need for future corrective or continual interventions. For example, in implant-based reconstructive approaches, the need for future monitoring of implant integrity with breast MRI should be discussed, as well as the anticipated lifespan of the implant and need for future exchange surgeries. A structured goals discussion should lay out a complication-threshold plan that designates pre-determined triggers for conservative vs. operative intervention, indexed to an adjuvant therapy timeline. Preparation for potential complications is not only an essential part of informed consent, but establishes realistic expectations for individual reconstructive outcomes, strengthening patient-provider trust and satisfaction.

## 8. Limitations

While the current library of literature on immediate breast reconstruction offers an exceptional volume of work that provides insight into technique-specific risks and clinical outcomes, high-quality evidence in several key areas is still lacking. There remains a paucity of evidence that directly compares patient-reported outcome measures and complication rates across each reconstructive modality, including a near absence of randomized controlled trials [54]. Evidence for hybrid approaches which combine free tissue transfer with implant placement and/or fat grafting also remains particularly sparse, with available studies often containing small cohort sizes that are primarily retrospective in nature [127,128,129]. Across all available literature, data on long-term follow-up results and patient-reported outcomes is limited, while the lack of universal classification systems for breast reconstruction complications creates heterogeneity that further hinders effective comparison [54,59,129]. Future studies are needed to address these knowledge gaps and advance understanding of immediate breast reconstruction techniques that will aid in critical comparison and strengthen an algorithmic approach to surgical planning.

## 9. Conclusions

Advancements in mastectomy strategies and the rise of innovative breast reconstruction strategies have expanded surgical options for patients while also increasing the complexity of perioperative decision-making. Combinations of mastectomy with reconstructive modalities are associated with unique complication profiles, some of which may disproportionately impact certain patient populations with potentially devastating consequences for reconstructive success. Demographic risk factors and patient comorbidities play an enormous role in dictating surgical outcomes, and the contribution of patient preference must ultimately be weighed against the safety and susceptibility of a patient to the postsurgical complications involved in a particular reconstructive approach. With a thorough understanding of these postoperative challenges and the patient-specific factors that may influence them, physicians can initiate more individualized surgical planning that helps each patient safely and effectively reach their reconstructive goals.

## Figures and Tables

**Table 1:** Summary of Complications Associated with Mastectomy and Immediate Breast Reconstruction. Complication profiles, incidence ranges, key risk factors, and management strategies organized by reconstructive modality.

Complication	Modality	Incidence	Key Risk Factors	Prevention and Management
UNIVERSAL COMPLICATIONS (All Mastectomy with IBR Types)				
Skin/NAC Necrosis	All (↑ in NSM)	Skin: 5–15% [14,15,16] NAC: 2.1–7% [17,18]	Smoking, high BMI, radiation, large breast volume, incision type, flap thickness	Conservative management; debridement (surgical or enzymatic); surgical revision for full-thickness necrosis
Contact Dermatitis/Skin Rash	All	Common; incidence not well-defined	Atopy, age, radiation history, prior topical reactions; surgical dressings (Tegaderm (3M, St. Paul, MN) or Dermabond (Ethicon Inc., Somerville, NJ)), skin prep agents	Remove offending agent; topical corticosteroids; most resolve in 2–4 weeks
Infection/Cellulitis	All (↑ implant-based)	Autologous: 7–8% [19,20] Implant: 5–14% [20,21,22]	Pre-op radiation/chemotherapy, high BMI, tobacco, hematoma, seroma, ADM use	PO/IV antibiotics; washout; explantation
Wound Dehiscence	All (↑ autologous)	Implant: 1–5.1% [23,24] DIEP: 3.5–17.4% [23,24,25,26]	Smoking, DM, obesity, suture line tension, incision type, prolonged OR time	Secondary intention healing; operative washout and reclosure for major or complex dehiscence
Seroma	All	Implant: 2.5–8.4% [27,28] Autologous: 4.6–21% [29,30,31]	Extent of breast/axillary dissection, lymph node removal, ADM, BMI, drain management	Closed suction drains; ROM exercises; percutaneous aspiration; culture if infection suspected
Hematoma	All	Autologous: 1.8–9.4% [31,32,33] Implant: 1–2.6% [32,34,35]	Anticoagulant/antiplatelet use, large implant size, coagulopathy, early postoperative activity	Monitor drain quality and quantity; operative evacuation to prevent mastectomy or flap ischemia; TXA (IV or topical) for prophylaxis
AUTOLOGOUS RECONSTRUCTION-SPECIFIC COMPLICATIONS				
Flap Failure (Total)	Autologous	0.5–5% [29,30,36]	Coagulopathy, small vessel caliber, obesity, smoking, prolonged OR time	Emergent return to OR; flap salvage; flap removal if unsalvageable; delayed reconstruction
Flap Failure (Partial)/Fat Necrosis	Autologous	Fat necrosis: 3.0–37.9% [37]	Inadequate perfusion, radiation (pre/post-op), flap weight, abdominal scarring, poor perforator selection	Supercharging (prophylaxis); Conservative treatment; hyperbaric O_2_; excision if symptomatic
Donor Site Wound Dehiscence	Autologous	DIEP: 3.5–14% [24,25,27]	Same as general; additional: abdominal closure tension, number of perforators harvested	Secondary intention; abdominal binder; surgical repair for deep fascial separation
Donor Site Seroma	Autologous (LD flap > abdominal)	LD flap highest among donor sites	Extent of harvest, dead space, drain management, BMI	Closed suction drains; aspiration
Donor Site Hernia/Abdominal Bulge	Autologous (DIEP)	0.7–4.9% [23,30,38] ↑ with ≥2 perforators; ↑ with lateral perforators	Obesity, age, perforator laterality, number of perforators harvested	PT for abdominal weakness; surgical repair with mesh for bulge/true hernia; APEX flap technique
IMPLANT-BASED RECONSTRUCTION–SPECIFIC COMPLICATIONS				
Capsular Contracture	Implant-based	10–25% [39,40] Variable; ↑ with textured implants and radiation	Radiation, textured implants, high BMI, mastectomy type (NSM vs. SSM), hematoma, infection	Capsulectomy; implant exchange; change in plane
Implant Rupture	Implant-based	10% to 15% of implants (within 10 to 15 years) [41] Saline: clinically obvious Silicone: often asymptomatic	Older implants, capsular contracture	Saline: early replacement (preserve pocket); Silicone: removal + capsulectomy; routine surveillance imaging after 5 years, then 2–3 years
Implant Extrusion	Implant-based (↑ prepectoral)	0.25–8% [42]	Prepectoral placement (reduced soft tissue coverage), post-mastectomy radiation, flap thickness	Explantation; delayed replacement or autologous conversion
BIA-ALCL	Implant-based (textured)	0.0025% to 0.18% [43] (textured implants); negligible for smooth implants	Macrotextured implants; chronic foreign body inflammatory response	En-bloc capsulectomy + implant removal; chemo/immunotherapy/ radiation for nodal/metastatic disease; ~2.8% mortality at 1 yr
BIA-SCC	Implant-based	Extremely rare (19 cases worldwide)	Not linked to specific implant characteristics; poorly understood	No standardized guidelines; ~48% mortality at 6 months
Animation Deformity	Implant-based (subpectoral)	75–100% (subpectoral) [44,45]	Subpectoral implant placement	Prepectoral conversion; accept if mild
Implant Malposition	Implant-based	~9.8% [46]	Pocket dissection plane, implant type, capsular contracture, body habitus	Internal/external sutures; dual-plane repositioning; ADM or capsular flap support; capsulorrhaphy/capsulectomy
Red Breast Syndrome (RBS)	Implant + ADM	Rare; exact incidence unclear	ADM use, post-mastectomy radiation, impaired lymphatic drainage, ADM DNA remnants (hypersensitivity)	Corticosteroids; antibiotics; ADM washout/removal for refractory cases; pre-implantation ADM saline wash

Abbreviations: IBR = immediate breast reconstruction; NSM = nipple-sparing mastectomy; NAC = nipple-areolar complex; SSM = skin-sparing mastectomy; ADM = acellular dermal matrix; LD = latissimus dorsi; DIEP = deep inferior epigastric perforator; TXA = tranexamic acid; DM = diabetes mellitus; BMI = body mass index; ROM = range of motion; OR = operating room; BIA-ALCL = breast implant-associated anaplastic large cell lymphoma; BIA-SCC = breast implant-associated squamous cell carcinoma; APEX = abdominal perforator exchange; PT = physical therapy. Incidence ranges are approximate and reflect heterogeneity across study designs, patient populations, follow-up durations, and outcome definitions.

**Table 2:** Patient-Specific Risk Stratification in Mastectomy and Immediate Breast Reconstruction. Patient characteristics, associated complication profiles, and mechanisms of risk.

Patient Characteristic Domain	Patient Characteristic	Associated Complications	Mechanism Influencing Complication Risk
Demographic Factors	Advanced age	Wound dehiscence, skin necrosis, infection, implant extrusion [26,27,57]	Thinning of skin and subcutaneous tissue paired with diminished cellular turnover compromises wound healing capacity; attenuated immune response increases infection susceptibility
Medical Comorbidities	Diabetes mellitus	Wound dehiscence, infection, skin/NAC necrosis, poor flap perfusion [24,29,58,59,60]	Impaired collagen production and integrity contribute to reduced wound tensile strength; hyperglycemia suppresses neutrophil function and antimicrobial defense
	Obesity (BMI ≥ 30 kg/m^2^)	Skin/NAC necrosis, infection, seroma, wound dehiscence, fat necrosis, abdominal hernia, flap failure [25,27,38,51,58,59,61]	Increased tension on skin flaps and closure lines; poorly vascularized adipose tissue susceptible to ischemia; larger dead space volumes promote fluid accumulation; abdominal wall mechanical stress elevated with visceral fat
	COPD	Wound dehiscence, abdominal hernia/bulge [27]	Chronic cough generates repeated surges in intra-abdominal pressure that stress fascial closures and donor site repairs
	Atopic conditions or history of allergies	Contact dermatitis, skin rash [62,63]	Atopic individuals may be predisposed to cutaneous hypersensitivity reactions to surgical dressings, prep agents, and topical antibiotics
	Coagulopathy	Hematoma, flap failure (thrombotic) [21,29]	Bleeding disorders and anticoagulant use elevate hematoma risk; conversely, thrombophilias predispose microvascular anastomoses to thrombosis and flap failure; both extremes complicate perioperative anticoagulation management
	Immunosuppression (e.g., autoimmune disease, transplant)	Infection, wound dehiscence [57]	Systemic immunosuppressants increase infection susceptibility through impaired neutrophil, macrophage, and T-cell functioning; corticosteroids directly inhibit collagen synthesis, compromising wound tensile strength
Oncologic Treatment History	Radiation therapy	Skin/NAC necrosis, infection, wound dehiscence, capsular contracture, implant extrusion, fat necrosis, flap failure [21,23,51,64,65,66]	Radiation induces endothelial dysfunction, microvasculature obliteration, and dermal fibrosis that impair tissue perfusion and wound healing; immunosuppressive effects of radiation reduce infection resistance; irradiated tissue is highly susceptible to implant-related complications
	Chemotherapy	Infection, wound dehiscence, seroma, poor flap perfusion [21,29,51]	Chemotherapy-induced myelosuppression impairs capacity to resist infections; cytotoxic agents disrupt endothelial cell regeneration needed for wound healing; chemotherapy-associated anemia reduces oxygen delivery to healing tissue
	Lymphedema/Axillary Lymph Node Dissection	Seroma, infection, wound dehiscence [29,51,67]	Impaired lymphatic drainage function creates conditions for seroma formation, contributing to accumulating fluid pressure that may threaten integrity of closure
Lifestyle Factors	Active or prior smoking history	Skin/NAC necrosis, infection, wound dehiscence [28,51,67,68]	Nicotine causes direct peripheral vasoconstriction, reducing perfusion to skin flaps and anastomosed free flaps; smoking-related immune suppression elevates infection risk
Anatomy and Body Habitus	Large breast size/ptosis	Skin necrosis, wound dehiscence, seroma [16,17,69]	Large breast volume creates greater skin flap tension and ischemic stretch on mastectomy flaps during dissection and postoperatively; ptosis necessitates longer incisions; larger reconstructions require greater implant volumes or flap mass, increasing mechanical stress on closures
	History of abdominal surgery	May preclude or complicate DIEP/TRAM flap use; fat necrosis, flap failure if autologous pursued [47,48,49]	Prior abdominal incisions (e.g., midline laparotomy, abdominoplasty, or liposuction) may disrupt perforator anatomy, damage the deep inferior epigastric vessels, and create scar tissue that limits tissue harvest; adhesions may complicate donor site dissection; altered fascial integrity from prior repair increases hernia risk at the donor site
	Availability of donor tissue	Precludes autologous reconstruction; may force implant-only approach with its associated risks [47,48,49]	Insufficient abdominal tissue volume (low BMI, prior lipectomy) may eliminate DIEP flap candidacy; lack of alternative donor sites (thigh, lumbar) forecloses autologous options entirely; patients directed toward implant-based reconstruction by donor site unavailability then carry standard implant-specific complication profiles

Abbreviations: BMI = body mass index; COPD = chronic obstructive pulmonary disease; NAC = nipple-areolar complex; DIEP = deep inferior epigastric perforator; TRAM = transverse rectus abdominis myocutaneous.

## Data Availability

No new data were created or analyzed in this study. Data sharing is not applicable to this article.
